# Deep Learning-Based Glaucoma Detection Using Clinical Notes: A Comparative Study of Long Short-Term Memory and Convolutional Neural Network Models

**DOI:** 10.3390/diagnostics15070807

**Published:** 2025-03-22

**Authors:** Ali Mohammadjafari, Maohua Lin, Min Shi

**Affiliations:** 1 School of Computing and Informatics, University of Louisiana at Lafayette, Lafayette, LA 70504, USA; ali.mohammadjafari1@louisiana.edu; 2 Department of Biomedical Engineering, Florida Atlantic University, Boca Raton, FL 33431, USA; mlin2014@fau.edu

**Keywords:** glaucoma detection, deep learning, LSTM, CNN, AI healthcare, clinical notes, fairness-aware modeling

## Abstract

**Background/Objectives:** Glaucoma is the second-leading cause of irreversible blindness globally. Retinal images such as color fundus photography have been widely used to detect glaucoma. However, little is known about the effectiveness of using raw clinical notes generated by glaucoma specialists in detecting glaucoma. This study aims to investigate the capability of deep learning approaches to detect glaucoma from clinical notes based on a real-world dataset including 10,000 patients. Different popular models are explored to predict the binary glaucomatous status defined from a comprehensive vision function assessment. **Methods:** We compared multiple deep learning architectures, including Long Short-Term Memory (LSTM) networks, Convolutional Neural Networks (CNNs), and transformer-based models BERT and BioBERT. LSTM exploits temporal feature dependencies within the clinical notes, while CNNs focus on extracting local textual features, and transformer-based models leverage self-attention to capture rich contextual information and feature correlations. We also investigated the group disparities of deep learning for glaucoma detection in various demographic groups. **Results:** The experimental results indicate that the CNN model achieved an Overall AUC of 0.80, slightly outperforming LSTM by 0.01. Both models showed disparities and biases in performance across different racial groups. However, the CNN showed reduced group disparities compared to LSTM across Asian, Black, and White groups, meaning it has the advantage of achieving more equitable outcomes. **Conclusions:** This study demonstrates the potential of deep learning models to detect glaucoma from clinical notes and highlights the need for fairness-aware modeling to address health disparities

## 1. Introduction

Glaucoma is a progressive optic neuropathy that ranks among the leading causes of irreversible blindness globally, impacting millions and imposing significant strain on healthcare systems [1,2]. The condition is characterized by damage to the optic nerve, frequently linked to elevated intraocular pressure, which results in visual field defects and potential vision loss if left untreated [1,3]. The disease’s gradual progression, particularly in its early stages, complicates detection, as initial signs are often subtle and may go unnoticed until substantial vision loss has occurred [4]. This underscores the critical need for the timely and precise prediction and classification of glaucoma to avert irreversible damage and enhance patient outcomes [5].

Current glaucoma diagnosis relies heavily on a combination of structural and functional tests, such as visual field testing, optical coherence tomography (OCT), and fundus imaging [6]. While effective, these methods require specialized equipment and are subject to limitations like inter-test variability and the need for skilled interpretation [3,6]. In contrast, clinical notes from routine ophthalmic visits contain rich, unstructured information that encompasses the patient history, examination findings, and progression details, which can be valuable for glaucoma detection [7]. Leveraging this unstructured resource through machine learning (ML) presents a great opportunity to enhance early detection and monitoring, especially in resource-limited settings where advanced imaging tools may not always be accessible [5,8,9].

Machine learning has demonstrated its potential in healthcare by providing automated, accurate, and scalable solutions for disease detection and risk prediction [10]. In this case, natural language processing (NLP) techniques allow for the analysis of unstructured clinical notes, extracting meaningful patterns that might be missed by human practitioners [11]. In this work, we explored the use of machine learning models, including recurrent neural networks such as Long Short-Term Memory (LSTM) networks, Convolutional Neural Networks (CNNs), and transformer models for the detection and classification of glaucoma from clinical notes. These models are well suited for understanding the temporal and contextual relationships within free-text data, making them powerful tools for analyzing clinical narratives and predicting disease outcomes [12].

Our study was based on the FairCLIP dataset [13], a vision–language medical dataset that includes clinical notes data. This dataset provides a unique opportunity to study glaucoma detection through multimodal analysis. The contribution of this study lies in implementing and benchmarking several deep learning models for glaucoma detection using clinical notes. These benchmark models (LSTM, CNN, transformer) were run individually to observe their differences and determine which model performs better for glaucoma detection. Using this dataset, the goal was to develop predictive models that not only classify glaucoma but also offer insights into disease progression across different demographic groups, addressing potential biases in diagnosis.

## 2. Related Works

The detection of glaucoma using machine learning has seen significant advancements, with researchers exploring various approaches involving imaging data, clinical notes, and hybrid models combining multiple data types [14,15]. One of the primary areas of study has been automated image analysis, where fundus photographs and optical coherence tomography (OCT) are leveraged to identify glaucoma using Convolutional Neural Networks (CNNs) [16].

While medical image-based deep learning approaches (e.g., CNNs for fundus images) have demonstrated strong glaucoma detection capabilities [13,17,18], these methods require high-quality imaging equipment, making them less accessible in some clinical settings. In contrast, clinical notes contain rich textual information about the patient history, symptoms, and diagnostic insights, yet remain underutilized for automated glaucoma detection. Our study shifts the focus from imaging-based methods to exploring deep learning models that can extract diagnostic signals from clinical notes.

This area of research involves the use of clinical notes, which are rich with unstructured information such as the patient history, examination findings, and treatment progression. Extracting meaningful data from these clinical notes using natural language processing (NLP) has been the focus of several studies [19]. Transformer-based models have been utilized to predict glaucoma progression from clinical ophthalmology notes that help to provide a deeper understanding of patient risk factors beyond what structured imaging data alone can provide. These approaches have demonstrated the potential for automated systems to support clinical decision-making, particularly in identifying high-risk patients who may require closer monitoring or surgical intervention [5].

Huang et al. developed a TRI-LSTM model to explore latent relationships among glaucoma biomarkers. Their approach modeled temporal relationships to improve the understanding of disease progression, adding interpretability to glaucoma detection. They highlighted the complexity of biomarker interrelations, which can be better understood through deep learning that simulates an expert’s diagnostic process [1].

Hu and Wang used transformer-based models, including RoBERTa and BioBERT, to predict glaucoma progression requiring surgery from clinical notes. Their models outperformed ophthalmologists in terms of F1 scores, highlighting the effectiveness of transformers in extracting insights from unstructured clinical data. They also demonstrated the benefits of combining clinical notes with structured data to enhance prediction accuracy, advancing the integration of clinical free text with machine learning for glaucoma care [20].

The development of machine learning models for glaucoma detection presents several challenges, such as the scarcity of labeled medical data and the need for effective preprocessing. Transfer learning has emerged as a common strategy to address these challenges, as demonstrated by Orlando et al. [17], who used pre-trained networks to extract relevant features without requiring large datasets specific to glaucoma. Additionally, models like the one proposed by Gheisari et al. [18] that incorporate both spatial and temporal data have shown promise in capturing the complex characteristics of glaucoma progression, particularly those related to vascular changes. Another significant challenge lies in integrating unstructured clinical notes into predictive models. The work of Hu and Wang [20] illustrates that transformer models can effectively parse and utilize free-text notes, while also pointing out the importance of further optimizing these models for domain-specific text. Moreover, datasets like those developed by Chen et al. [21] are crucial for enabling more effective NLP applications in ophthalmology, helping bridge the gap between imaging and text data. The current body of work highlights the importance of leveraging diverse data types—including imaging, clinical notes, and structured EHR data—to build robust models for glaucoma detection. Advances in transfer learning, hybrid deep learning architectures, and NLP offer promising avenues for further improving the accuracy and generalizability of these models, with the ultimate goal of enabling the earlier diagnosis and personalized treatment of glaucoma patients [22].

Despite these advancements, prior studies have not systematically compared different deep learning architectures (e.g., LSTM vs. CNNs) for clinical note-based glaucoma detection. Additionally, existing models often overlook fairness considerations, leading to potential biases in diagnostic predictions across demographic groups. This study addressed these gaps by benchmarking LSTM and CNN models on a real-world dataset and analyzing performance disparities among racial groups to assess fairness-aware glaucoma detection.

## 3. Methodology

In this study, we developed deep learning models to classify the presence of glaucoma from clinical notes, leveraging both Long Short-Term Memory (LSTM) networks and Convolutional Neural Networks (CNNs), and then compared them with transformer-based models like BERT and BioBERT. The goal was to evaluate different architectures and techniques for feature extraction from unstructured text, determining the most effective approach for glaucoma detection.

### 3.1. Data Preparation and Preprocessing

The dataset used for this study consisted of clinical notes from electronic health records (EHRs), along with demographic attributes such as age, gender, ethnicity, and language. These notes contained detailed physician observations, symptoms, diagnostic impressions, and follow-up recommendations. A sample clinical note is as follows:

These free-text notes are tokenized and converted into numerical sequences using a tokenizer. The processed text was then passed to deep learning models for glaucoma classification.

**Figure 1:**
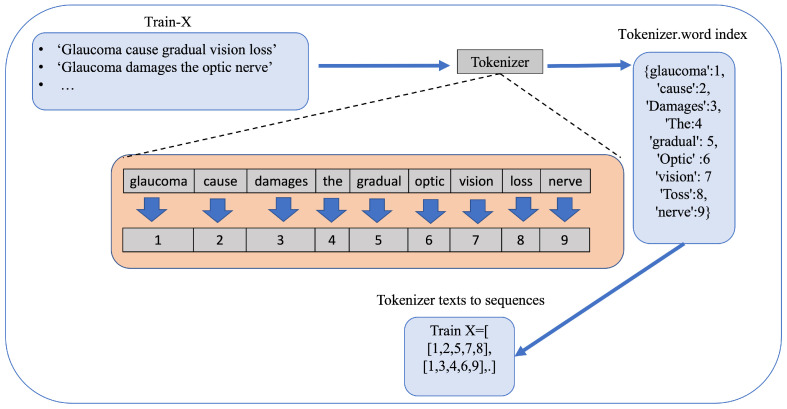
Tokenizer workflow. The process of converting clinical notes into numerical sequences using a tokenizer, where each word is mapped to a unique integer for the model input.

A tokenizer was fitted on the text column of the dataset to convert the clinical notes and GPT-4-generated summaries into sequences of tokens. These token sequences were padded to a fixed maximum length (set at 100) to ensure uniformity across the input data. The clinical notes, which contained unstructured information about the patient history, examination findings, and treatment, were treated as the primary input for model training. The tokenizer workflow is shown in Figure 1. The target column, “glaucoma”, was preprocessed by converting its values to binary labels: ‘yes’ was converted to 1, and ‘no’ was converted to 0, representing the presence or absence of glaucoma.

### 3.2. Model Interpretability

To enhance model interpretability, we applied Grad-CAM to identify key tokens that contributed to glaucoma prediction. Given the challenge of applying Grad-CAM directly to text-based models, we first computed Grad-CAM importance scores for each clinical note.

We then aggregated the importance scores across all clinical notes, computing the average importance score for each word based on its occurrence frequency-weighted contribution. Aggregated importance analysis across all notes highlighted the most frequently important words. This approach allowed us to visualize which words were most indicative of glaucoma risk, ensuring transparency in model decision-making.

### 3.3. Model Architecture

#### 3.3.1. Long Short-Term Memory (LSTM) Model

**Figure 2:**
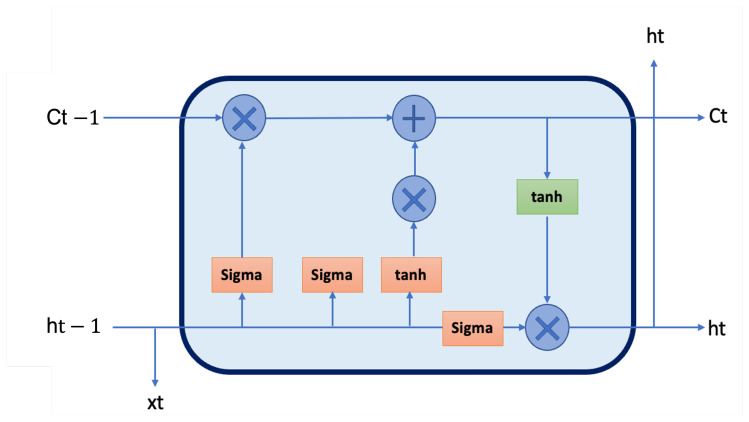
The structure of a long short-term memory (LSTM) cell [23].

Recurrent neural networks (RNNs) are commonly used for text-based tasks due to their ability to model sequential dependencies. However, standard RNNs struggle with long-range dependencies due to vanishing gradients. To address this, we employed a Long Short-Term Memory (LSTM) network, which maintains long-term dependencies in clinical narratives through its gating mechanisms. The structure of LSTM can be found in Figure 2.

In this study, the LSTM model processed tokenized clinical notes to capture temporal patterns that may indicate glaucoma progression. The model first embedded each token into a dense vector representation via an embedding layer. These embeddings were then passed through two stacked LSTM layers, where the first layer captured short-term dependencies and the second layer refined representations by integrating long-term contextual information. To prevent overfitting and improve generalization, we applied dropout regularization in both LSTM layers, along with L1/L2 weight regularization in the final dense layer. The output layer employed a sigmoid activation function, producing a probability score for glaucoma presence. Compared to CNNs, which capture local phrase-based patterns, LSTM is advantageous in processing sequential dependencies within clinical texts, such as tracking the progression of optic nerve damage or monitoring intraocular pressure changes over time.

#### 3.3.2. Convolutional Neural Network (CNN) Model

**Figure 3:**
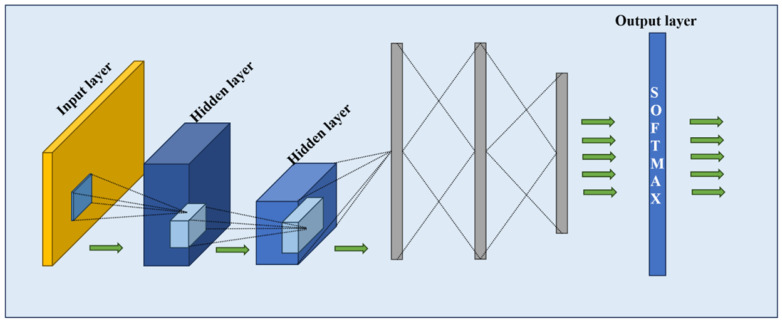
The Convolutional Neural Network architecture diagram for the classification problem [26].

Convolutional Neural Networks (CNNs) are widely used in NLP tasks due to their ability to capture local patterns and hierarchical structures in text [24,25]. The model architecture of a CNN is shown in Figure 3. In this study, we employed a CNN model to extract key features from clinical notes, focusing on local dependencies that may indicate glaucoma presence. The model began with an embedding layer that converted tokenized clinical notes into dense vector representations. These embeddings were processed through two convolutional layers, each applying a kernel with a size of 5 with ReLU activation to detect significant n-gram patterns relevant to medical terminology.

To prevent excessive feature dimensionality, max pooling layers followed each convolutional layer, reducing spatial complexity while retaining critical patterns. The extracted feature maps were then aggregated using a fully connected dense layer, followed by a sigmoid activation function, which output the probability of a glaucoma diagnosis. This architecture enabled the CNN to identify key clinical phrases such as “optic nerve damage” or “increased intraocular pressure”, which are essential for diagnosis. Compared to LSTM, which captures sequential dependencies, CNNs provide a complementary approach by emphasizing local contextual cues in text.

CNN models provide advantages for glaucoma detection from clinical notes, especially in capturing the spatial and local dependencies within the text. By using weight-sharing and local connectivity, CNNs are capable of learning complex feature representations with fewer parameters, reducing overfitting and improving efficiency. The use of pooling further enhanced the model’s robustness to small variations in the text, contributing to better generalization during real-world application.

## 4. Dataset and Experimental Setup

### 4.1. Clinical Notes Dataset

We used a public dataset called FairCLIP developed by Harvard researchers, as described in [27]. FairCLIP contains 10,000 clinical notes paired with fundus images from 10,000 glaucoma patients who received glaucoma services at Massachusett Eye and Ear. We only used the 10,000 clinical notes for our study to investigate the performance of glaucoma detection using clinical notes only. These clinical notes contain rich textual information recorded during patient visits. The dataset also includes demographic attributes such as age, gender, race, ethnicity, language, and marital status, which can influence glaucoma diagnosis and model fairness.

The clinical notes have an average length of 925 characters, capturing detailed descriptions of the patient history, examination findings, and disease progression. The dataset is relatively balanced, with 50.48% of cases labeled as glaucoma-positive and 49.52% labeled as glaucoma-negative. In this dataset, 76.9% of data are for the White race, 14.9% for the Black race, and 8.19% for the Asian race.

### 4.2. Experimental Setup

To assess the performance of the deep learning models, the following standard classification metrics were employed:

- Area Under the Receiver Operating Characteristic Curve (AUC-ROC): Measured the model’s ability to distinguish between glaucoma and non-glaucoma cases.
- Accuracy: Proportion of correctly classified cases.
- Sensitivity (Recall): Measured the percentage of glaucoma cases correctly identified by the model.
- Specificity: Measured the percentage of correctly classified non-glaucoma cases.
- Confusion Matrix: Provided insight into false positive and false negative rates.

### 4.3. Model Parameters

In this study, the LSTM model consisted of two layers, with the first LSTM layer containing 32 hidden units and the second layer containing 16 hidden units. Each layer applied L1 and L2 regularization to prevent overfitting, along with batch normalization and a dropout rate of 0.3 to enhance model generalization. The model was trained using the Adam optimizer with a learning rate of 0.001 for 14 epochs and a batch size of 32.

Regarding the CNN model, the model designed to extract key textual features comprised two convolutional layers. The first convolutional layer had 64 filters, while the second had 32 filters, both utilizing a kernel size of 5. ReLU activation was applied throughout the network, followed by max pooling layers to reduce dimensionality. A global max pooling layer preceded the fully connected layer, which consisted of 32 units with ReLU activation. Similarly to the LSTM model, the CNN was trained using the Adam optimizer with a learning rate of 0.001 for 14 epochs and a batch size of 32. Regularization techniques, including dropout with a rate of 0.3, were implemented to mitigate overfitting.

## 5. Results and Discussion

### 5.1. Token Importance Analysis Using Grad-CAM

**Figure 4:**
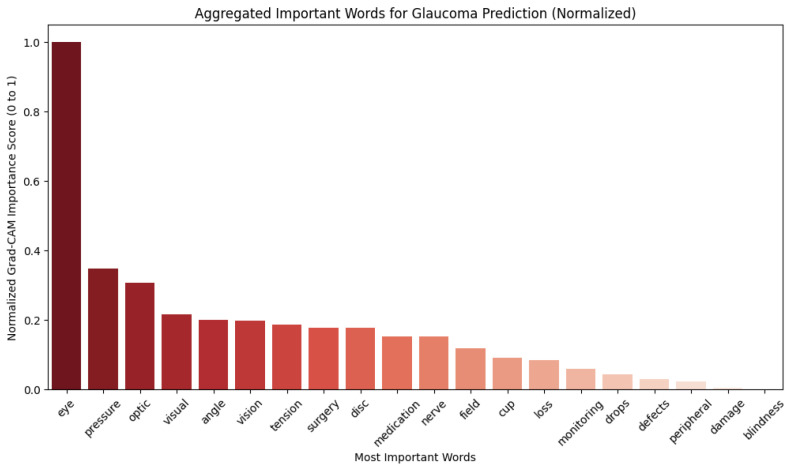
Aggregated important words for glaucoma prediction.

The first analysis, the results of which are shown in Figure 4, showed the most important words in predicting glaucoma from a single clinical note, demonstrating that terms related to optic nerve damage, vision loss, and intraocular pressure had high Grad-CAM scores.

**Figure 5:**
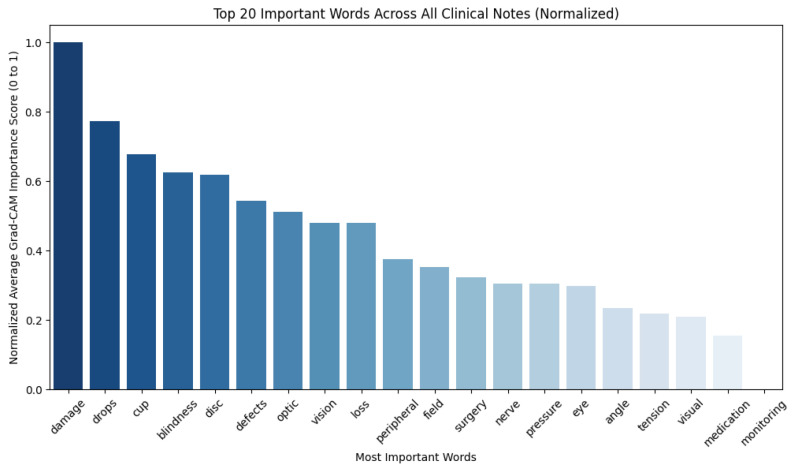
Top 20 important words across all clinical notes.

The second analysis, the results of which are shown in Figure 5, aggregated token importance scores across all clinical notes, revealing that words such as “damage”, “drops”, “cup”, and “blindness” consistently contributed most to the model’s decision-making.

The results align with clinical knowledge, as these terms are highly relevant to glaucoma diagnosis and management. This analysis improves the interpretability of our CNN-based prediction model, ensuring that predictions are driven by medically meaningful terms.

### 5.2. LSTM Model Performance

In this study, clinical notes from FairCLIP datasets [13] were used for training the models. The LSTM model was used to detect glaucoma from clinical notes by leveraging the temporal relationships inherent in textual data.

**Figure 6:**
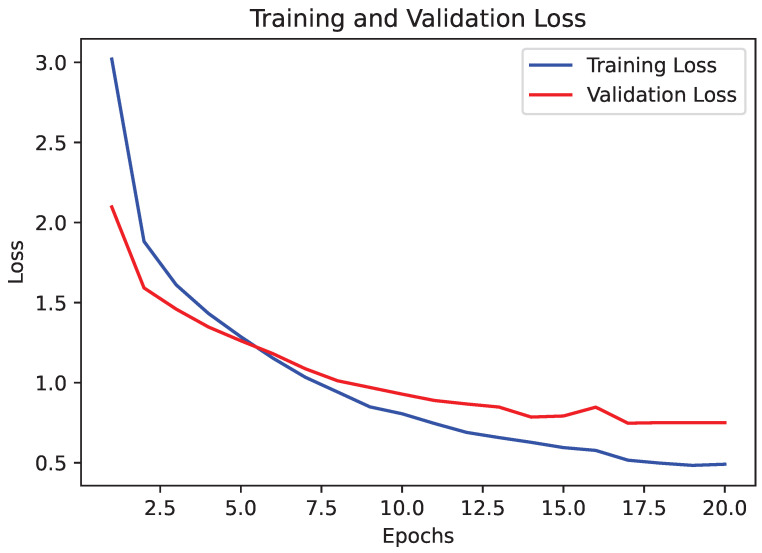
Training and validation loss for LSTM.

To mitigate overfitting in the LSTM model, we incorporated multiple regularization techniques. We applied dropout layers (0.5 and 0.6) to prevent the co-adaptation of neurons and enhance generalization. To further improve model robustness, L1 and L2 regularization were used in both LSTM and dense layers, preventing excessive weight growth. Additionally, an early-stopping mechanism was implemented, monitoring the validation loss and halting training when performance degradation was detected. These strategies collectively enhanced the model’s ability to generalize while maintaining optimal predictive accuracy. The training and validation loss plot, Figure 6, shows a steady decrease over 14 epochs, indicating effective learning. However, while regularization techniques such as dropout and early stopping helped control overfitting, the slight divergence between the training and validation losses in later epochs suggests residual overfitting, potentially limiting the model’s generalizability.

**Figure 7:**
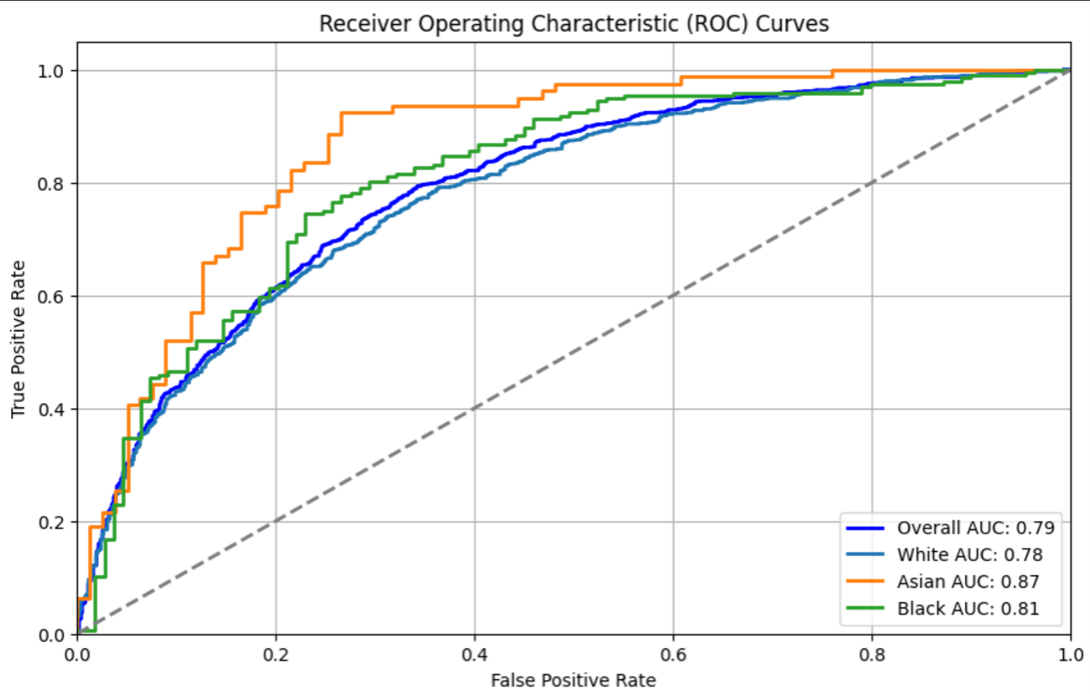
AUC for LSTM.

The LSTM model’s Receiver Operating Characteristic (ROC) curves, as depicted in Figure 7, show the model’s overall performance across different demographic groups. The model achieved an Overall AUC of 0.79, with an AUC of 0.78 for the White group, 0.87 for the Asian group, and 0.81 for the Black group. These results indicate variability in model performance across demographic groups, with the highest performance observed for the Asian group.

**Figure 8:**
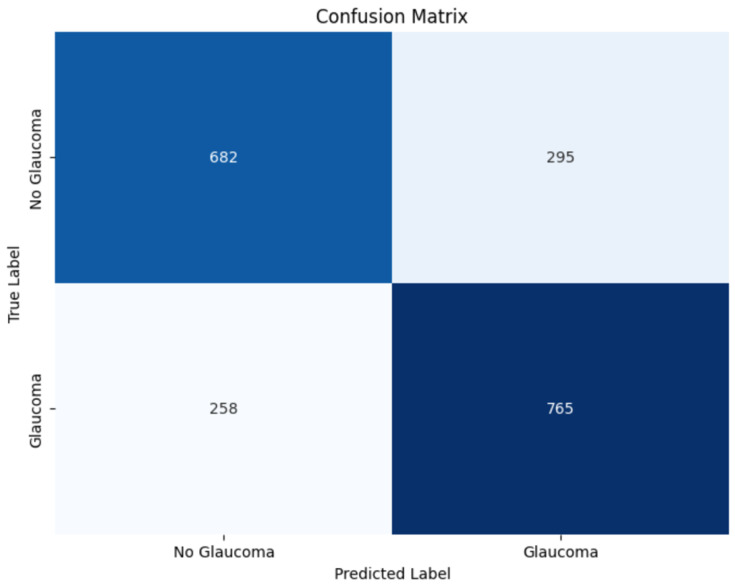
Confusion matrix for LSTM.

The confusion matrix, shown in Figure 8, provides further insight into the model’s classification capability. The LSTM model correctly identified 682 instances without glaucoma (true negatives) and 765 cases with glaucoma (true positives), while incorrectly classifying 295 non-glaucoma cases as glaucoma (false positives) and 258 glaucoma cases as non-glaucoma (false negatives). This translates to a modest sensitivity but suggests some challenges with specificity, which may impact clinical applicability by producing a relatively high number of false positives.

### 5.3. CNN Model Performance

**Figure 9:**
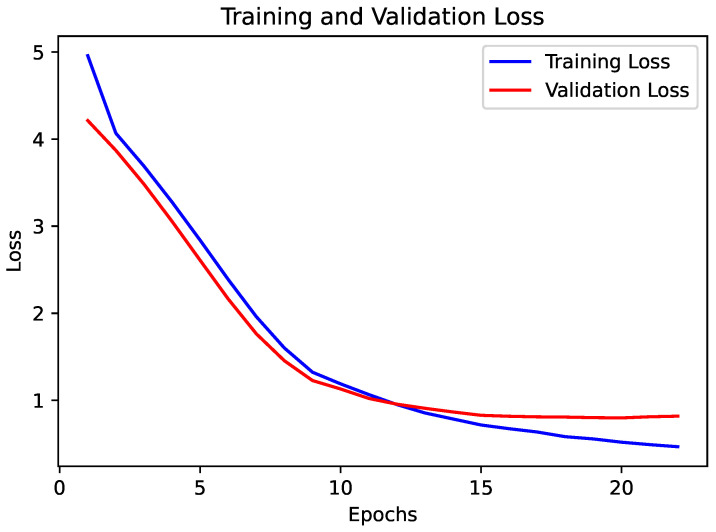
Training and validation loss for CNN.

The CNN was also evaluated as a benchmark model for glaucoma detection. The CNN architecture was designed to capture local spatial dependencies in the clinical text data, which is particularly useful in identifying crucial indicators of glaucoma that may be distributed across the text. The training and validation loss plot, shown in Figure 9, shows a consistent decline, and the validation loss follows the training loss closely, suggesting effective learning without significant overfitting.

**Figure 10:**
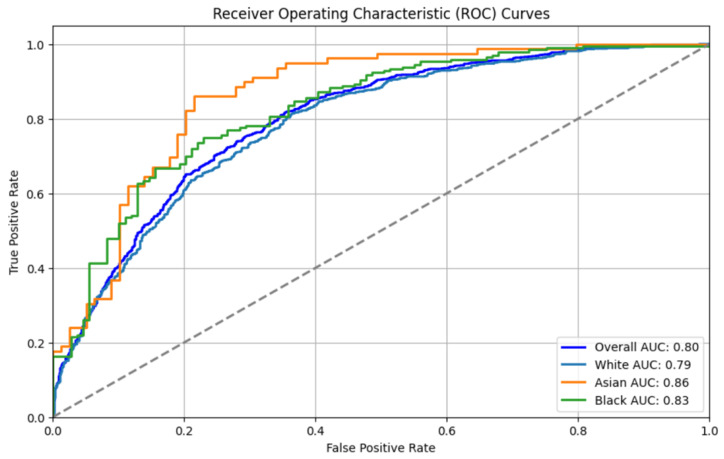
AUC for CNN.

The ROC curves for the CNN model, shown in Figure 10, show an improvement over the LSTM model, with an Overall AUC of 0.80. The AUCs for the White, Asian, and Black groups were 0.79, 0.86, and 0.83, respectively. The CNN model demonstrated a better balance in performance across the demographic groups, reflecting an improvement in fairness compared to the LSTM model. This may be attributed to the convolutional layers’ ability to effectively extract key features and mitigate biases present in the textual data.

**Figure 11:**
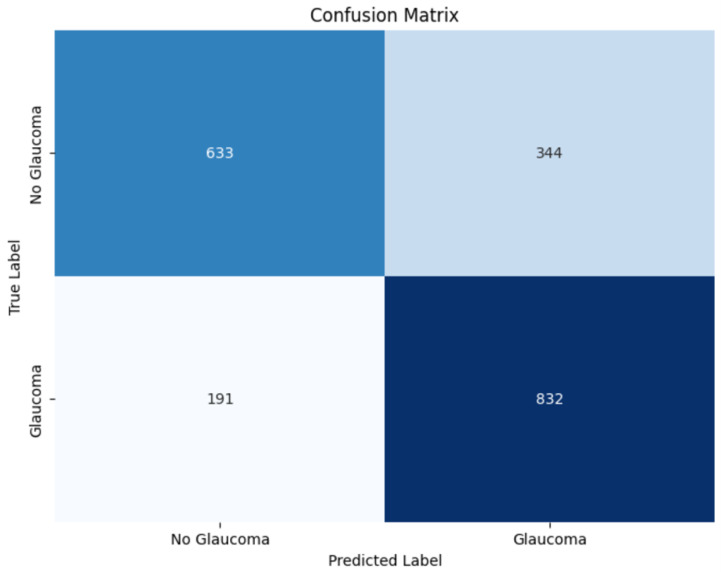
Confusion matrix for CNN.

The confusion matrix, shown in Figure 11, reveals that the CNN model identified 633 true negatives and 832 true positives, with 344 false positives and 191 false negatives. Compared to the LSTM model, the CNN produced fewer false negatives, indicating improved sensitivity in glaucoma detection. The higher true positive count and lower false negative rate imply that the CNN was more adept at identifying glaucoma cases, making it a more reliable model for real-world screening applications.

### 5.4. Comparative Analysis

Comparing the LSTM, CNN, and transformer-based models reveals important differences in their performance and learning capabilities. Transformer models such as BERT and BioBERT have been increasingly applied in clinical NLP tasks due to their ability to capture contextual dependencies more effectively. The CNN model demonstrated superior performance across most metrics, including a higher Overall AUC and improved balance across demographic groups. This can be attributed to the convolutional layers’ ability to capture both local dependencies and important features that may be distributed across the clinical notes, which are not learned as well by the LSTM model.

The LSTM model, while capable of modeling sequential dependencies, was more prone to overfitting, as evidenced by the divergence in the training and validation losses. This may suggest that LSTM’s reliance on sequential relationships was less effective for the type of clinical text data used in this study, which benefited more from feature extraction using convolutional operations.

The CNN model’s improved sensitivity, as shown by the higher true positive rate and lower false negative rate, makes it more favorable for clinical applications where missing a positive case could have significant adverse effects on patient outcomes. However, the relatively high false positive rate for both models remains a limitation, as it may lead to unnecessary follow-ups or interventions.

Table 1 compares the performance of our CNN and LSTM models with results from prior studies, including those using text-based models and transformer-based approaches such as BERT and BioBERT. Our CNN model outperformed prior text-based models and was competitive with transformer-based models like BERT-Base and BioBERT. While multimodal models performed slightly better in terms of the AUC, our proposed models provided strong predictive performance without requiring additional structured data.

### 5.5. Fairness-Aware Learning and Adversarial Debiasing

To mitigate potential bias in the model predictions, we implemented adversarial debiasing, training separate adversarial networks for different genders and races. The adversarial networks were designed to remove demographic bias while maintaining high precision for glaucoma prediction. LSTM was considered as a base model.

Table 2 summarizes the model performance before and after applying adversarial debiasing:

The results indicated that applying adversarial debiasing slightly reduced the AUC but improved fairness across demographic subgroups. The gap between the demographic AUC scores decreased, demonstrating a reduction in bias. Future work could explore additional fairness-aware learning techniques such as equalized odds postprocessing and demographically stratified training.

### 5.6. Computational Efficiency Analysis

To evaluate the feasibility of deploying our AI models in clinical settings, we evaluated their computational efficiency in terms of floating-point operations (FLOPs) and their inference latency. Both the LSTM and CNN models were analyzed regarding their computational cost and suitability for real-time deployment. The results are summarized in Table 3.

These results confirm that while the LSTM model has a higher computational cost, it remains feasible for hospital server deployment. Meanwhile, the CNN model is computationally more efficient, achieving faster inference times, making it suitable for real-time Clinical Decision Support Systems (CDSSs) and potential edge device deployment. Future optimizations, such as model quantization and pruning, could further enhance the performance of both architectures.

## 6. Conclusions and Discussion

The Grad-CAM analysis provided insights into which clinical terms drove model predictions. Our findings demonstrate that the model prioritized medically relevant terms, such as “damage”, “pressure”, and “optic”. This confirms that the CNN-based model learns patterns aligned with clinical decision-making. Future work can extend this approach by integrating attention-based mechanisms or using transformers (e.g., ClinicalBERT) to improve the token-level explainability. Our CNN and LSTM models achieved AUCs of 80% and 79%, respectively, which are higher than those of prior text-based models and comparable to those of transformer-based architectures. These results suggest that even without multimodal fusion, deep learning models trained on clinical notes can be effective for glaucoma prediction.

Integration into Clinical Decision Support Systems (CDSSs) is a crucial step toward the real-world deployment of AI models in healthcare. To enhance clinical adoption, our approach can be incorporated into existing CDSS platforms by providing real-time insights for glaucoma detection from clinical notes. By integrating it directly into electronic health record (EHR) systems, the model can assist ophthalmologists by highlighting critical risk factors and aiding in decision-making. Additionally, model outputs can be structured to offer explainable recommendations, improving trust and transparency in AI-assisted diagnoses. Future work should explore API-based integration with hospital IT systems, enabling seamless deployment in clinical workflows while ensuring regulatory compliance and patient data security.

The disparity in performance across demographic groups for both models highlights an ongoing challenge in developing equitable machine learning models for healthcare. The higher AUC observed for the Asian group in both models suggests potential biases in data representation, which could stem from imbalances in the dataset or inherent differences in clinical note characteristics. Addressing these biases is crucial to ensure fair and equitable diagnostic outcomes across all patient groups.

Future work could focus on enhancing model generalizability by incorporating fairness-aware learning techniques, such as demographic parity regularization or domain adaptation methods. Additionally, integrating hybrid approaches that combine both LSTM and CNN architectures could leverage the strengths of both models, potentially improving both the feature extraction and temporal representation capabilities.

## Figures and Tables

**Table 1:** Comparison of model performance for glaucoma prediction across different approaches.

Model	AUC	Accuracy	F1 Score
Our CNN	80%	79.6%	0.757
Our LSTM	79%	76.7%	0.735
Text-based model [5]	41%	39%	0.53
Multimodal model [5]	73%	70%	0.74
BERT-Base [20]	76%	75%	0.45
BioBERT [20]	70%	68%	0.42

**Table 2:** Impact of adversarial debiasing on model performance.

Metric	Baseline Model	Gender-Debiased Model	Race-Debiased Model
Overall AUC	0.79	0.78	0.77
AUC (Male)	0.78	0.79	0.78
AUC (Female)	0.80	0.79	0.78
AUC (White)	0.78	0.78	0.79
AUC (Black)	0.81	0.79	0.78
AUC (Asian)	0.87	0.86	0.85

**Table 3:** Computational efficiency comparison of LSTM and CNN models.

Metric	LSTM Model	CNN Model
FLOPs	1,020,273	672,097
Batch Processing Time (32 samples)	0.5985 s	0.1854 s
Single-Inference Time	0.5887 s	0.1846 s
Total Test Set Inference Time	1.80 s	0.38 s

## Data Availability

The data presented in this study are openly available in https://ophai.hms.harvard.edu/datasets/harvard-gdp1000 (accessed on 17 March 2025).

## References

[1] Huang C., Shen J., Luo Q., Kooner K., Lee T., Liu Y., Zhang J. (2024). Latent Relationship Mining of Glaucoma Biomarkers: A TRI-LSTM based Deep Learning. arXiv.

[2] Kim S.J., Cho K.J., Oh S. (2017). Development of machine learning models for diagnosis of glaucoma. PLoS ONE.

[3] Akter N., Fletcher J., Perry S., Simunovic M.P., Briggs N., Roy M. (2022). Glaucoma diagnosis using multi-feature analysis and a deep learning technique. Sci. Rep..

[4] Pakka N., Rauniyar K., Dangal S., Chaulagain R. (2023). Evaluation of Network Intrusion Detection with Feature Selection using Random Forest and Deep Neural Network. KEC J. Sci. Eng..

[5] Jalamangala Shivananjaiah S.K., Kumari S., Majid I., Wang S.Y. (2023). Predicting near-term glaucoma progression: An artificial intelligence approach using clinical free-text notes and data from electronic health records. Front. Med..

[6] Hung K.H., Kao Y.C., Tang Y.H., Chen Y.T., Wang C.H., Wang Y.C., Lee O.K.S. (2022). Application of a deep learning system in glaucoma screening and further classification with colour fundus photographs: A case control study. BMC Ophthalmol..

[7] Tian Y., Wen C., Shi M., Afzal M.M., Huang H., Khan M.O., Luo Y., Fang Y., Wang M. Fairdomain: Achieving fairness in cross-domain medical image segmentation and classification. Proceedings of the European Conference on Computer Vision.

[8] Lin W.C., Chen A., Song X., Weiskopf N.G., Chiang M.F., Hribar M.R. (2024). Prediction of multiclass surgical outcomes in glaucoma using multimodal deep learning based on free-text operative notes and structured EHR data. J. Am. Med. Inform. Assoc..

[9] Ajorloo S., Jamarani A., Kashfi M., Kashani M.H., Najafizadeh A. (2024). A systematic review of machine learning methods in software testing. Appl. Soft Comput..

[10] Chan K., Lee T.W., Sample P.A., Goldbaum M.H., Weinreb R.N., Sejnowski T.J. (2002). Comparison of machine learning and traditional classifiers in glaucoma diagnosis. IEEE Trans. Biomed. Eng..

[11] Hossain E., Rana R., Higgins N., Soar J., Barua P.D., Pisani A.R., Turner K. (2023). Natural language processing in electronic health records in relation to healthcare decision-making: A systematic review. Comput. Biol. Med..

[12] Mohammadjafari A., Maida A.S., Gottumukkala R. (2024). From natural language to sql: Review of llm-based text-to-sql systems. arXiv.

[13] Luo Y., Shi M., Khan M.O., Afzal M.M., Huang H., Yuan S., Tian Y., Song L., Kouhana A., Elze T. Fairclip: Harnessing fairness in vision-language learning. Proceedings of the IEEE/CVF Conference on Computer Vision and Pattern Recognition.

[14] Barros D.M., Moura J.C., Freire C.R., Taleb A.C., Valentim R.A., Morais P.S. (2020). Machine learning applied to retinal image processing for glaucoma detection: Review and perspective. Biomed. Eng. Online.

[15] Li F., Wang Z., Qu G., Song D., Yuan Y., Xu Y., Gao K., Luo G., Xiao Z., Lam D.S. (2018). Automatic differentiation of Glaucoma visual field from non-glaucoma visual filed using deep convolutional neural network. BMC Med. Imaging.

[16] Khunger M., Choudhury T., Satapathy S.C., Ting K.C. (2019). Automated detection of glaucoma using image processing techniques. Emerging Technologies in Data Mining and Information Security: Proceedings of IEMIS 2018, Volume 3.

[17] Orlando J.I., Prokofyeva E., del Fresno M., Blaschko M.B. Convolutional neural network transfer for automated glaucoma identification. Proceedings of the 12th International Symposium on Medical Information Processing and Analysis.

[18] Gheisari S., Shariflou S., Phu J., Kennedy P.J., Agar A., Kalloniatis M., Golzan S.M. (2021). A combined convolutional and recurrent neural network for enhanced glaucoma detection. Sci. Rep..

[19] Wang S.Y., Tseng B., Hernandez-Boussard T. (2022). Deep learning approaches for predicting glaucoma progression using electronic health records and natural language processing. Ophthalmol. Sci..

[20] Hu W., Wang S.Y. (2022). Predicting glaucoma progression requiring surgery using clinical free-text notes and transfer learning with transformers. Transl. Vis. Sci. Technol..

[21] Chen J.S., Lin W.C., Yang S., Chiang M.F., Hribar M.R. (2022). Development of an open-source annotated glaucoma medication dataset from clinical notes in the electronic health record. Transl. Vis. Sci. Technol..

[22] Milon T.I., Rauniyar K., Furman S., Orthi K.H., Wang Y., Raghavan V., Xu W. (2024). Representing and Quantifying Conformational Changes of Kinases and Phosphatases Using the TSR-Based Algorithm. Kinases Phosphatases.

[23] Mohammadjafari A. (2024). Comparative Study of Bitcoin Price Prediction. arXiv.

[24] Naghshnejad P., Theis Marchan G., Olayiwola T., Kumar R., Romagnoli J. (2024). Graph-Based Modeling and Molecular Dynamics for Ion Activity Coefficient Prediction in Polymeric Ion-Exchange Membranes. Ind. Eng. Chem. Res..

[25] Zhai Z., Zhang X., Fang F., Yao L. (2023). Text classification of Chinese news based on multi-scale CNN and LSTM hybrid model. Multimed. Tools Appl..

[26] Freeman I., Roese-Koerner L., Kummert A. Effnet: An efficient structure for convolutional neural networks. Proceedings of the 2018 25th IEEE International Conference on Image Processing (ICIP).

[27] Luo Y., Shi M., Tian Y., Elze T., Wang M. Harvard glaucoma detection and progression: A multimodal multitask dataset and generalization-reinforced semi-supervised learning. Proceedings of the IEEE/CVF International Conference on Computer Vision.

